# First evidence of Crimean‐Congo haemorrhagic fever virus circulation in Bosnia and Herzegovina

**DOI:** 10.1002/vms3.781

**Published:** 2022-03-08

**Authors:** Lejla Satrovic, Adis Softic, Almedina Zuko, Aida Kustura, Amira Koro, Sejla Goletic, Edin Satrovic, Francisco Llorente, Elisa Pérez‐Ramírez, Jasmin Omeragic, Jasna Salkic, Amer Alic, Miguel Angel Jiménez‐Clavero, Teufik Goletic

**Affiliations:** ^1^ Veterinary Faculty University of Sarajevo Sarajevo Bosnia and Herzegovina; ^2^ Centro de Investigación en Sanidad Animal (CISA), INIA‐CSIC Valdeolmos Spain; ^3^ Department of Pathology University Clinical Center Tuzla Tuzla Bosnia and Herzegovina; ^4^ Faculty of Medicine University of Tuzla Tuzla Bosnia and Herzegovina; ^5^ Centro de Investigación Biomédica en Red de Epidemiologia y Salud Pública (CIBERESP) Madrid Spain

**Keywords:** Bosnia and Herzegovina, CCHFV, serology, sheep, tick‐borne diseases

## Abstract

**Background:**

Crimean–Congo haemorrhagic fever (CCHF) is a widespread tick‐borne zoonosis with reported detection of virus and/or virus‐specific antibodies from over 57 countries across Africa, Asia, Europe and the Middle East and is endemic in the Balkans. Detection of Crimean–Congo Haemorrhagic Fever Virus (CCHFV) antibodies in domestic ruminants has been important in providing initial evidence of virus circulation and in localising CCHFV high‐risk spots for human infection.

**Objectives:**

The present study investigated the possible exposure of sheep to CCHFV in Bosnia and Herzegovina (B&H).

**Methods:**

To investigate the presence of anti‐CCHFV antibodies in sheep, all sera (*n* = 176) were tested using multi‐species double antigen enzyme‐linked immunosorbent assay (ELISA). Reactive sera were further complementary tested by adapted commercial indirect immunofluorescence assay (IFA) using FITC‐conjugated protein G instead of anti‐human immunoglobulins.

**Results:**

CCHFV specific antibodies were detected in 17 (9.66%) animals using ELISA test. All negative sera were determined as negative by both tests, while 13 out of 17 ELISA‐positive reactors were also determined as unambiguously positive by IFA test. The age group with the highest proportion of seropositive rectors were the oldest animals.

**Conclusions:**

This is the first report of anti‐CCHFV antibodies in sheep from B&H providing the evidence of CCHFV circulation in the country's sheep population. So far, these findings indicate the circulation of the virus in the westernmost region of the Balkans and point to the potential CCHFV spread further out of this endemic area.

## Abstract 2

Crimean Congo Hemorrhagic Fever Virus (CCHFV) infection is endemic in Balkans. A serological survey of 176 sheep was conducted to investigate the circulation to CCHFV in Bosnia and Herzegovina. CCHFV specific antibodies were detected in 17 (9.66%) animals providing the early evidence of CCHFV circulation in the country's sheep population.

## 1 INTRODUCTION

Crimean–Congo haemorrhagic fever virus (CCHFV; family *Nairoviridae*, genus *Orthonairovirus*), the causative agent of Crimean–Congo haemorrhagic fever (CCHF) has been recognised as an emerging pathogen of great importance with significant potential as a weapon of biological warfare (Kuhn et al., 2020; WHO, 2021). CCHF is a widespread tick‐borne zoonosis with reported detection of virus and/or virus‐specific antibodies from over 57 countries across Africa, Asia, Europe and the Middle East (Bente et al., 2013). The majority of human infections have an asymptomatic clinical course; however the development of severe clinical forms of the disease in infected people is not such a rare event and should not be neglected (Bente et al., 2013; Portillo et al., 2021). The transmission occurs through the bite of infected ticks; however, blood and other body fluids of infected animals and human patients represent an additional source of infection (Gunes et al., 2009). The virus is maintained in an enzootic cycle between ixodid ticks, predominantly *Hyalomma* genus that serves as vectors and reservoirs with transovarial, transstadial, venereal and non‐viremic (co‐feeding) transmission among them, and wild and domestic vertebrates (Turell, 2007). Domestic ruminants, despite experiencing transient viremia, remain clinically asymptomatic and pose latent sources of infection for ticks and humans (Bente et al., 2013). Despite the well‐known CCHF endemicity in the western Balkan region (Messina et al., 2015), there has been no evidence of CCHFV circulation in Bosnia and Herzegovina (B&H). Furthermore, there are no control measures or surveillance programs for CCHF put in place. Detection of CCHFV antibodies in domestic animals has been important in providing initial evidence of virus circulation and in localising CCHFV high‐risk spots for human infection (Spengler et al., 2016). Moreover, it is well known that the first reports of the disease in some countries coincided with the introduction of effective control measures (Spengler et al., 2019). Therefore, our study aimed to investigate the possible exposure of sheep to CCHFV in B&H.

## 2 MATERIAL AND METHODS

**FIGURE 1:**
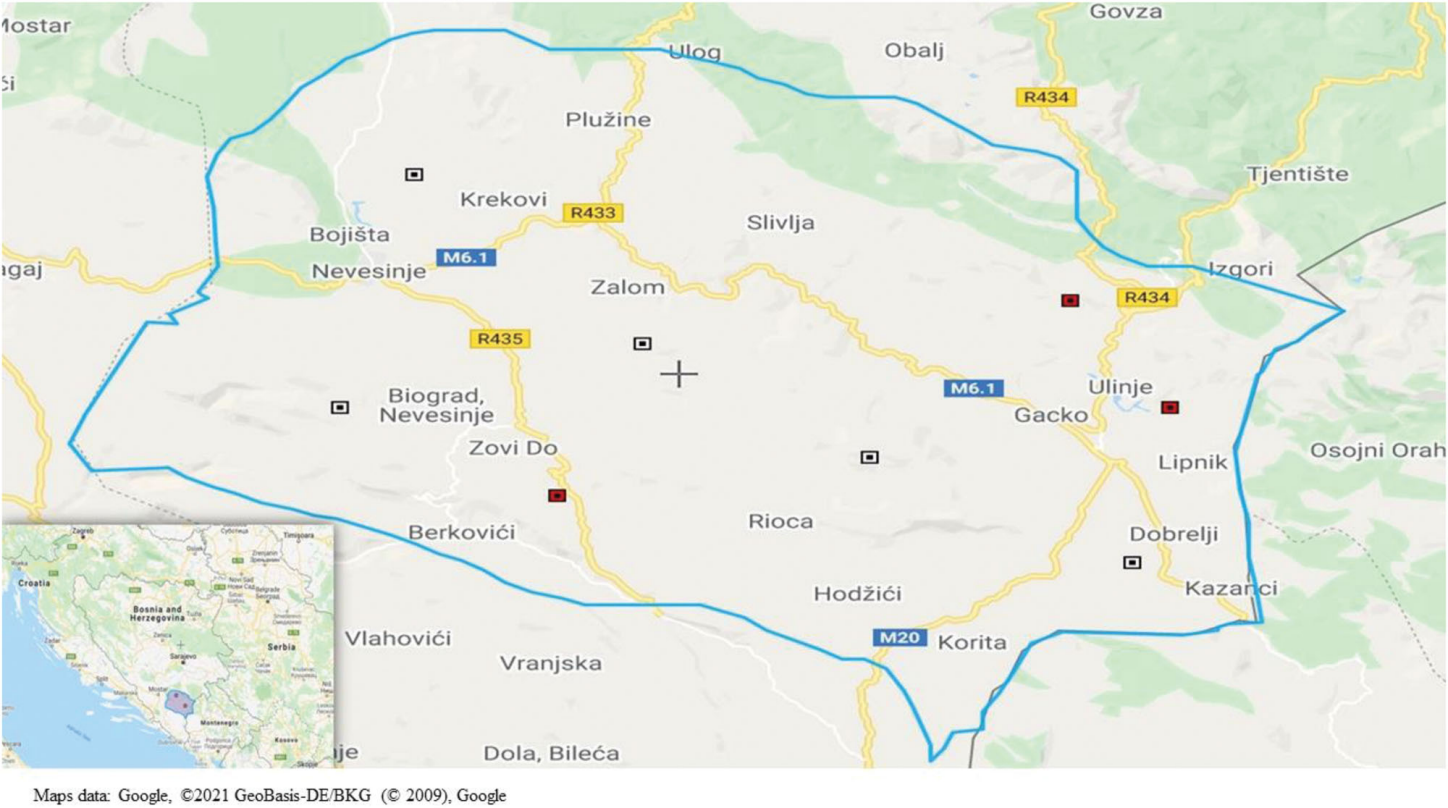
Selected municipalities in the southeastern part of Bosnia and Herzegovina (left corner) and the distribution of selected farms in two previously selected municipalities. Seropositive individual animals/reactors to CCHFV were found in three farms (red squares), while there were no seropositive individual animals in five farms (white squares).

Considering the aim of the study, the most suitable study area was the southeastern part of B&H due to its specific geographic position, that is, the proximity to CCHFV endemic regions in Serbia and Kosovo (Figure 1). This area is characterised by the moderate continental climate from the north and the Mediterranean climate from the south (Statistical Yearbook of Republika Srpska, 2018), known as a natural habitat for ixodid ticks (Omeragic, 2011), that is, vectors of the disease. Two municipalities Gacko and Nevesinje with associated local communities were selected for the study. Geographical and bioclimatic features of the selected study area are given in Table 1.

**TABLE 1:** Characteristics of the selected study localities and sheep flocks in southwestern Bosnia and Herzegovina

Location/municipality	Region of B&H	Number of flocks (total number of animals)	Bioclimatic status	Latitude (N)	Longitude (E)	Mean altitude (m)
Gacko	Southeast	4 (86)	Moderate continental – pastures	43°10'1.99"	18°32'7.01"	940
Nevesinje	Southeast	4 (90)	Moderate continental – pastures	43°15'31.00"	18°06'47.99"	860

Given that selection of the study area was convenient, the sampling strategy was purposive, organised as risk‐based two‐stage sampling. In the first stage, the target population was all sheep flocks located in the previously selected municipalities. Afterwards, the source population was drawn from the available lists of all sheep flocks submitted by the State Veterinary Office of B&H, as well as local veterinary or agricultural organisations, while in the second stage, the final study sample consisted of individual female animals from selected flocks. All individual animals from each selected flock were randomly selected using computer‐generated random numbers, and the number of individual animals per flock depended on the flock size.

A total of eight flocks were selected and 176 sera samples were collected from individual animals with no clinical signs of the disease. The average age of selected individual animals was three years. The sampling procedure took place in the period May–June 2018. Blood samples (5 ml) were collected from the jugular vein (*v. jugularis*) of each selected individual animal, using sterile needles and plain vacutainer tubes. After collection, the samples were transported to the Laboratory for molecular‐genetic and forensic investigations where samples were allowed to stand overnight at room temperature to obtain the serum. Optionally, the samples were centrifuged at 3000 × *g* for 5 min. Serum was pipetted into cryovials and stored at −20°C until being tested.

This study was conducted under the Law on Animal Protection and Welfare of BH (‘Official Gazette BH’ issue number 316/09). Samples were collected after acquiring permission from study participants. All farmers/owners who participated in this provided their written consent from all sheep owners expressing willingness to participate in the study. This study was submitted to and approved by the relevant country's Ethics Committee.

The collected serum samples were tested in duplicate by two independent laboratories using a double‐antigen ELISA kit (ID Screen^®^ CCHF Double Antigen Multi‐species, IDVet) following the manufacturer's instructions. The ELISA kit has high sensitivity and specificity: 98.9% (95% CI: 96.8–99.8) and 100% (95% CI: 99.8–100), respectively, and is optimised for the detection of total antibodies against CCHFV in serum and plasma samples of cattle, sheep, goats and other susceptible species (Sas et al., 2018). A second serological technique was used to confirm the results obtained: all samples were tested with an adapted version of the commercial indirect immunofluorescence assay (IFA; CCHFV Mosaic 2, Euroimmun, Lübeck, Germany) using FITC‐conjugated protein G instead of anti‐human immunoglobulins as secondary detection step (unpublished data).

## 3 RESULTS

Out of 176 serum samples examined by ELISA, 17 (9.66%) were found to be positive for CCHFV. The ELISA test results from both laboratories were congruent, without any significant difference and therefore presented as mean values of results obtained from both testing laboratories (Table 3). The mean optical density (OD) value in the ELISA for positive sera was 1.151 (*n* = 17; Max, 2.024; Min, 0.382; SD = 0.4669). The OD for negative sera ranged from 0.012 to 0.163. Regarding the geographic distribution, 14 (16.27%) positive reactors were found in two sheep flocks in the municipality of Gacko while 3 positive reactors (3.33%) were found in one sheep flock in the municipality of Nevesinje (Figure 1). All tested animals were indigenous purebred Pramenka or its crossbreeds. Except for age, all individual characteristics of reactors were unknown due to the lack of written flock records. The age group with the highest proportion of seropositive rectors were the oldest animals (Table 2). While IFA confirmed all negative samples as negative, 13 out of 17 ELISA positive samples yielded IFA positive results. The remaining four samples gave inconclusive results (Table 3). All IFA results in which it was not possible to unambiguously assess the fluorescence of both target proteins (CCHFV‐GPC and CCHFV‐N) or on one of them while the other was negative were declared as inconclusive results. However, these samples were not further retested.

**TABLE 2:** The distribution of CCHFV seropositive animals represented over the age group in the sheep population in Bosnia and Herzegovina

Age group (years)	Number of seropositive/number of tested (% ± SE)
0–2	2/64 (3.12 ± 2.17)
3–4*	9/80 (11.25 ± 3.53)
5–6	6/32 (18.75 ± 6.89)

* Some age records were based on estimation

**TABLE 3:** Comparative presentation of results of two serological tests (ELISA and IFA) used for investigation of CCHFV in sheep population in Bosnia and Herzegovina

	ID Screen^®^ CCHF DA ELISA			IFA TEST
Sample no.	Optical density	S/P % ratio	POS/NEG	POS/NEG
1	0.908	102.02	POS	POS
2	2.024	215.96	POS	POS
3	1.502	168.76	POS	POS
4	1.023	114.94	POS	POS
5	1.759	197.64	POS	DBT
6	1.264	142.02	POS	POS
7	1.29	144.94	POS	POS
8	1.091	122.58	POS	POS
9	0.382	54.61	POS	DBT
10	1.759	197.64	POS	POS
11	1.717	192.92	POS	POS
12	1.694	190.34	POS	POS
13	1.361	152.92	POS	DBT
14	1.282	144.04	POS	POS
15	1.913	214.94	POS	POS
16	1.231	138.31	POS	DBT
17	1.239	139.21	POS	POS

## 4 DISCUSSION

Thus far, there is no scientific data of CCHFV circulation in animals or vectors, nor have the cases of the disease been reported in humans in B&H. Hence, to the best of our knowledge, this study provides early evidence of CCHFV circulation in animals in B&H. The exposure to CCHFV, that is, the evidence of specific antibodies in the sheep population, was confirmed by serological tests. The discrepancy between ELISA and IFA test may be due to known differences in the detectability of sheep immunoglobulins by both kits: while ELISA is reactive to both IgG and IgM, the latter would not be expected to react in the adapted IFA. Indeed, this outcome may reflect different infection stages among sampled animals (Dowall et al., 2012). In this study, older animals were more likely to be seropositive for CCHFV in comparison with younger animals (Table 2). This observation coincides with a recent study of age dependent CCHFV seroprevalences in Mauritanian livestock (Schulz et al., 2021). The simple explanation of this phenomenon is faced in the possibility that older animals are exposed to CCHFV‐infected competent vectors longer than younger animals, and thus becoming infected with the virus. Considering genetic and antigenic close relationships with other Orthonairoviruses, the possibility that antibodies directed against them might interfere with current CCHFV serological assays should not be neglected (Kalkan‐Yazıcı et al., 2021). In case that such antibodies led to false‐positive CCHFV test results, the distribution and prevalence of CCHFV could be rather overestimated, especially in regions where other Othonairoviruses are prevalent (Hartlaub et al., 2021). However, considering that there is no scientific evidence of the introduction and circulation of other Orthonairoviruses in the Balkan region, the possibility of serological cross‐reactions is substantially low.

Possible factor that could have contributed to the emergence of seropositive individual animals in the southeastern parts of the country is a favourable climatic condition for the life cycle of ticks, especially from the genus *Hyalomma*. In addition to vectors, the specific geographic position of the investigated area, that is, relative proximity to endemic regions in eastern neighbouring countries (Serbia, Kosovo and Montenegro), could partially contribute to the CCHFV emergence in B&H. This can be explained by the nomadic and semi‐nomadic pastoralism and the possibility of illegal cross‐border transport and trade of animals from the CCHFV endemic regions to the naïve sheep population in B&H. In such a situation and given the fact that there is no CCHFV surveillance program in B&H, the import of potentially infected animals and vectors cannot be completely ruled out. Based on our results, favourable climatic and ecological conditions with the proven presence of competent vectors in B&H (Omeragic, 2011) as well as reports of CCHF/CCHFV detection in the rest of the Balkan region (Dreshaj et al., 2016), it is reasonable to assume that there is a possibility for CCHFV circulation in B&H as well. However, taking into account a relatively small study area and the number of investigated animals, the results presented here warrant further widespread investigation to get a real picture of the circulation of CCHFV in the whole country. In line with this, our further research will focus on gathering additional serologic as well as molecular evidence of CCHFV circulation in this but also in other regions of B&H, both in ticks and in susceptible vertebrates. At the same time, it is necessary to increase the level of caution and awareness in the medical and veterinary sectors at the national level due to enhanced risk of infection for humans in close contact with the potentially infected animals, following the principles of the ‘One Health’ concept. Moreover, it is of paramount importance to develop an effective CCHFV surveillance program for humans, animals and vectors in B&H. Last but not least, our results indicate the circulation of the virus in the westernmost region of the Balkans so far and point to the potential CCHFV spread further out of this endemic area. Along with recent reports of established circulation of CCHFV genotypes in Spain (Negredo et al., 2021), data reported herein suggest the narrowing of the gap between the well‐known endemic regions and Western Europe. The threat of possible continuous expansion and circulation of the virus over Central Europe and further throughout the continent should not be disregarded.

## AUTHOR CONTRIBUTIONS

Lejla Satrovic: conceptualisation; data curation; investigation; methodology; writing – original draft; writing – review & editing. Adis Softic: conceptualisation; formal analysis; methodology; visualisation; writing – review & editing. Almedina Zuko: investigation; resources. Aida Kustura: investigation; methodology. Amira Koro: investigation. Sejla Goletic: data curation; investigation; writing – original draft. Edin Satrovic: investigation; resources. Francisco Llorente: formal analysis; methodology; validation. Elisa Pérez‐Ramírez: formal analysis; methodology; validation. Jasmin Omeragic: investigation; resources. Jasna Salkic: data curation; methodology. Amer Alic: data curation; writing – review & editing. Teufik Goletic: conceptualisation; data curation; funding acquisition; investigation; methodology; project administration; supervision; validation; writing – original draft; writing – review & editing.

## ETHICAL APPROVAL

The authors confirm that the ethical policies of the journal, as noted on the journal's author guidelines page, have been adhered to. No ethical approval was required in this study.

## CONFLICT OF INTEREST

All authors declare no conflict of interest.

### PEER REVIEW

The peer review history for this article is available at https://publons.com/publon/10.1002/vms3.781.

## Data Availability

The data that support the findings of this study are available from the corresponding author upon reasonable request.

## References

[vms3781-bib-0001] Bente, D. A. , Forrester, N. L. , Watts, D. M. , McAuley, A. J. , Whitehouse, C. A. , & Bray, M. (2013). Crimean‐Congo hemorrhagic fever: History, epidemiology, pathogenesis, clinical syndrome and genetic diversity. Antiviral Research, 100(1), 159–189. 10.1016/j.antiviral.2013.07.006 23906741

[vms3781-bib-0002] Dowall, S. D. , Richards, K. S. , Graham, V. A. , Chamberlain, J. , & Hewson, R. (2012). Development of an indirect ELISA method for the parallel measurement of IgG and IgM antibodies against Crimean‐Congo haemorrhagic fever (CCHF) virus using recombinant nucleoprotein as antigen. Journal of Virological Methods, 179(2), 335–341. 10.1016/j.jviromet.2011.11.020 22155577

[vms3781-bib-0003] Dreshaj, S. , Ahmeti, S. , Ramadani, N. , Dreshaj, G. , Humolli, I. , & Dedushaj, I. (2016). Current situation of Crimean‐Congo hemorrhagic fever in Southeastern Europe and neighboring countries: A public health risk for the European Union? Travel Medicine and Infectious Disease, 14(2), 81–91. 10.1016/j.tmaid.2016.03.012 27044611

[vms3781-bib-0004] Gunes, T. , Engin, A. , Poyraz, O. , Elaldi, N. , Kaya, S. , Dokmetas, I. , Bakir, M. , & Cinar, Z. (2009). Crimean‐Congo hemorrhagic fever virus in high‐risk population, Turkey. Emerging Infectious Diseases, 15(3), 461–464. 10.3201/eid1503.080687 19239765 PMC2681111

[vms3781-bib-0005] Hartlaub, J. , Daodu, O.B. , Sadeghi, B. , Keller, M. , Olopade, J. , Oluwayelu, D. , & Groschup, M.H. (2021). Cross‐reaction or co‐infection? serological discrimination of antibodies directed against Dugbe and Crimean‐Congo hemorrhagic fever orthonairovirus in Nigerian cattle. Viruses, 13(7):1398. 10.3390/v13071398 34372604 PMC8310240

[vms3781-bib-0006] Kalkan‐Yazıcı, M. , Karaaslan, E. , Çetin, N. S. , Hasanoğlu, S. , Güney, F. , Zeybek, Ü. , & Doymaz, M. Z. (2021). Cross‐reactive anti‐nucleocapsid protein immunity against Crimean‐Congo hemorrhagic fever virus and hazara virus in multiple species. Journal of Virology, 95(7), e02156‐20. 10.1128/JVI.02156-20 PMC8092682 33441341

[vms3781-bib-0007] Kuhn, J. H. , Adkins, S. , Alioto, D. , Alkhovsky, S. V. , Amarasinghe, G. K. , Anthony, S. J. , Avšič‐Županc, T. , Ayllón, M. A. , Bahl, J. , Balkema‐Buschmann, A. , Ballinger, M. J. , Bartonička, T. , Basler, C. , Bavari, S. , Beer, M. , Bente, D. A. , Bergeron, É. , Bird, B. H. , Blair, C. , Blasdell, K. R. ,… Zhou, X. (2020). 2020 taxonomic update for phylum Negarnaviricota (Riboviria: Orthornavirae), including the large orders Bunyavirales and Mononegavirales. Archives of Virology, 165(12), 3023–3072. 10.1007/s00705-020-04731-2 32888050 PMC7606449

[vms3781-bib-0008] Messina, J. P. , Pigott, D. M. , Golding, N. , Duda, K. A. , Brownstein, J. S. , Weiss, D. J. , Gibson, H. , Robinson, T. P. , Gilbert, M. , William Wint, G. R. , Nuttall, P. A. , Gething, P. W. , Myers, M. F. , George, D. B. , & Hay, S. I. (2015). The global distribution of Crimean‐Congo hemorrhagic fever. Transactions of the Royal Society of Tropical Medicine and Hygiene, 109(8), 503–513. 10.1093/trstmh/trv050 26142451 PMC4501401

[vms3781-bib-0009] Negredo, A. , Sánchez‐Ledesma, M. , Llorente, F. , Pérez‐Olmeda, M. , Belhassen‐García, M. , González‐Calle, D. , Sánchez‐Seco, M.P. , & Jiménez‐Clavero, M.A. (2021). Retrospective identification of early autochthonous case of Crimean‐Congo hemorrhagic fever, Spain, 2013. Emerging Infectious Diseases, 27(1). 10.3201/eid2706.204643. PMC8153886 34013861

[vms3781-bib-0010] Omeragic, J. (2011). Ixodid ticks in Bosnia and Herzegovina. Experimental and Applied Acarology, 53(3), 301–309. 10.1007/s10493-010-9402-8 20967487

[vms3781-bib-0011] Portillo, A. , Palomar, A. M. , Santibáñez, P. , & Oteo, J. A. (2021). Epidemiological aspects of Crimean‐Congo hemorrhagic fever in Western Europe: What about the future? Microorganisms, 9(3), 649. 10.3390/microorganisms9030649 33801015 PMC8003855

[vms3781-bib-0012] Sas, M. A. , Comtet, L. , Donnet, F. , Mertens, M. , Vatansever, Z. , Tordo, N. , Pourquier, P. , & Groschup, M. H. (2018). A novel double‐antigen sandwich ELISA for the species‐independent detection of Crimean‐Congo hemorrhagic fever virus‐specific antibodies. Antiviral Research, 151, 24–26. 10.1016/j.antiviral.2018.01.006 29330092

[vms3781-bib-0013] Schulz, A. , Barry, Y. , Stoek, F. , Ba, A. , Schulz, J. , Haki, M. L. , Sas, M. A. , Doumbia, B. A. , Kirkland, P. , Bah, M. Y. , Eiden, M. , & Groschup, M. H. (2021). Crimean‐Congo hemorrhagic fever virus antibody prevalence in Mauritanian livestock (cattle, goats, sheep and camels) is stratified by the animal's age. PLoS Neglected Tropical Diseases, 15, e0009228. 10.1371/journal.pntd.0009228 33844691 PMC8081336

[vms3781-bib-0014] Spengler, J. R. , Bergeron, É. , & Rollin, P. E. (2016). Seroepidemiological studies of Crimean‐Congo hemorrhagic fever virus in domestic and wild animals. PLoS Neglected Tropical Diseases, 10(1), e0004210. 10.1371/journal.pntd.0004210 26741652 PMC4704823

[vms3781-bib-0015] Spengler, J. R. , Bergeron, É. , & Spiropoulou, C. F. (2019). Crimean‐Congo hemorrhagic fever and expansion from endemic regions. Current Opinion in Virology, 34, 70–78. 10.1016/j.coviro.2018.12.002 30660091 PMC6497153

[vms3781-bib-0016] Statistical Yearbook of Republika Srpska 2018 [Internet document]. 2018 [cited 2021 Oct 25]. Retrieved from: www.rzs.rs.ba/static/uploads/bilteni/godisnjak/2018/StatistickiGodisnjak_2018_WEB.pdf;

[vms3781-bib-0017] Turell, M.J. (2007). Role of ticks in the transmission of Crimean‐Congo hemorrhagic fever virus. In: O. Ergonul & C. A. Whitehouse (Eds.). Crimean‐Congo hemorrhagic fever: A global perspective (pp. 143–154). Dordrecht: Springer.

[vms3781-bib-0018] World Health Organization . Prioritizing diseases for research and development in emergency contexts. WHO [Internet document]. 2021 [cited 2021 Feb 17]. Retrieved from: https://www.who.int/activities/prioritizing‐diseases‐for‐research‐and‐development‐in‐emergency‐contexts.

